# No time to grieve: Inuit loss experiences and grief practices in Nunavik, Quebec

**DOI:** 10.1177/13634615221135423

**Published:** 2022-11-07

**Authors:** Shawn Renee Hordyk, Mary Ellen Macdonald, Paul Brassard, Looee Okalik, Louisa Papigatuk

**Affiliations:** 1 École de travail social, 229169 Université du Québec à Montréal Faculté des sciences humaines, Canada; 2 Faculty of Dentistry, 5620 McGill University, Canada; 3 Division of Clinical Epidemiology – McGill University Health Centre, 5620 McGill University, Canada; 4 Community Knowledge Holder, Montreal, Canada

**Keywords:** caregivers, community, grief, Inuit, loss, Nunavik

## Abstract

This article presents an overview of past and current grief rituals and practices and existing grassroots and institutional initiatives seeking to address the complex, prolonged, and traumatic grief experienced by many Inuit living in Quebec. While conducting a study seeking to identify the strengths, resources, and challenges for Nunavik's Inuit communities related to end-of-life care, results emerged concerning how family caregivers’ grief related to the dying process was compounded by the sequelae of historic loss experiences (e.g., losses related to Canada's federal policies, including residential schools, forced relocations, and dog slaughters) and by present loss experiences (e.g., tragic and sudden deaths in local communities). To better support caregivers, an understanding of these grief experiences and a vision of bereavement care inclusive of community mobilization efforts to develop bereavement training and support is needed. We conclude with a discussion of a community capacity approach to bereavement care.

## Introduction

With global aging populations, end-of-life (EOL) care has become the focus of increased attention. Caregivers providing EOL care in Nunavik, the Inuit region of northern Quebec, are increasing in number as population growth has resulted in higher numbers of ageing Inuit and those dying prematurely from terminal illnesses. In Nunavik, the cancer rate is 30% higher than that of the general Quebec population and respiratory diseases are 75% higher (Statistics Canada, 2013). Simultaneously, the region has experienced higher mortality rates due to traumatic deaths including accidents, suicides, and disappearances (Inuit Tapiriit Kanatami, 2016). Statistics Canada reports that Nunavimmiut (the people of Nunavik) are three times more likely to die due to unintentional injuries than the rest of the Quebec population, and 7.5 times more likely to die by self-inflicted injury (Statistics Canada, 2013).

In addition to experiencing the anticipatory grief that occurs in caring for a loved one at the end of life (Cheung et al., 2018; Nielsen et al., 2017), caregivers in Nunavik often experience grief due to prior losses. In the context of a six-year project exploring the implementation of community-based palliative care services in Canadian First Nation's communities, Kelley et al. (2018) identified lack of grief support for families, local health providers, and the community at large as a significant barrier to care. Compounding this suffering, the technical aspects of EOL care have become more complex, and grief related to losses is increasingly present in the community. The task of providing care has become significantly demanding, both physically and emotionally. While listening to family caregivers discuss their experiences of EOL care provision, we continually heard how the grief related to prior death and loss experiences affected their ability to care for those going through the process of dying (Hordyk et al., 2017b). We know little of the extent to which multiple grief experiences and mourning-related beliefs and practices impact palliative care provision in Nunavik. We believe that addressing the prevalence and distinctiveness of Nunavimmiut grief experiences, beliefs, and practices is essential to support the mental health and well-being of caregivers and to maintain their capacity to provide care.

### The context of end-of-life care in Nunavik

Nunavik, one of Canada's four Inuit regions, includes 14 fly-in communities located within the northern borders of the province of Quebec ranging from populations of 225 to 2,700 persons; four communities have over 1,000 habitants. Thirty-seven percent of Inuit in Nunavik are under 14 years of age. Less than 5% are over 60 years old. Inuktitut is the primary language spoken. The medical support offered to families providing EOL care depends on the size of the community. EOL care in Nunavik communities is usually provided by family, community members, local health workers and interpreters, onsite nurses, and either visiting or resident physicians (Hordyk et al., 2017a). Each Nunavik community has a health center staffed by a minimum of two nurses; larger communities have a designated home care nurse and one or more resident physicians. Two communities offer limited 24-h inpatient care. As most of the Nunavik population identify Inuktitut as their first language, nurses and physicians rely heavily on local interpreters to provide care, a challenging task for both (Hordyk et al., 2017b).

The grief that persons experience preceding and following the death of a loved one is accentuated by the challenging context in which care is offered. These complications are familiar to many Indigenous communities where logistic, demographic, geographic, environmental, institutional, interpersonal, socio-cultural, linguistic, and historical considerations shape palliative care (Caxaj et al., 2018; Hordyk et al., 2016, 2017a). Families providing care are often simultaneously confronted by the reduced physical and relational capacities of patients nearing the end of life and increased economic stress related to loss of income or costs related to care (Lobb et al., 2010; Payne et al., 1999). There are few respite services available for Nunavik patients and caregivers (Hordyk et al., 2017a). While efforts are made to provide the formal and informal support structures needed for patients and families to remain in communities, patients may need to be flown far from family and community to one of the two hospitals in Nunavik or to a hospital in Montreal (Hordyk et al., 2017a). When sent to the South for care, patients are often accompanied by one escort, and a small number of additional family members can be provided plane tickets by the local health board when the patient is close to death. Costs for travel of extended family can be prohibitive, with regular flights between North and South costing between CA$2,100 and CA$3,500 per round trip. However, Nunavik's primary airline, Air Inuit, provides compassionate travel fares for bereaved persons: a reduction of 75% for Inuit attending funerals outside of their communities and reductions of 50% and 75% for friends and family members of seriously ill patients.

### Grief, mourning, and bereavement

The terms *grief*, *mourning*, and *bereavement* are often used interchangeably in the literature to describe a person's reaction to death. Grief can be attributed to an experience of loss that is non-death-related, and mourning is often used to describe a public response to death. Grief theorists emphasize the multifaceted, unpredictable, and complex aspects of the grief, one that is highly individualized and influenced by factors such as sex (Doka & Martin, 2011), age (Walter & McCoyd, 2015), culture (Stroebe & Schut, 1998), and the circumstances of death (Nader, 1997; Parkes & Prigerson, 2013).

Zisook and Shear (2013) have described grief as a process within which persons vacillate between accepting and resisting the reality of death and within which persons may experience widely varying emotional, cognitive, social, and behavioral reactions to the death. Though feelings of grief may come and go, grief can eventually become integrated into the life and identity of the bereaved person such that they can continue to function and develop ongoing relationships. In certain circumstances, however, a normal range of emotions experienced by a griever can become debilitating and cause enduring suffering and functional impairments. These grief experiences have been characterized via an array of terminologies and diagnostic criteria such as *disenfranchised grief* (Doka, 1989), *pathological grief* (Horowitz et al., 1993), *complicated grief* (Prigerson et al., 1995; Shear et al., 2011), *traumatic grief* (Prigerson et al., 1997), and *prolonged grief* (Boelen & Prigerson, 2013). In terms of medical diagnostic categories, the DSM-5 (American Psychiatric Association, 2013) has chosen the term “persistent complex bereavement related disorder” to categorize this, and the ICD-11 (World Health Organization, 2018) “prolonged grief disorder.” Criteria in both instances are similar, though the DSM-5 suggests 12 months and the ICD-11 six months as the period in which debilitating grief-related symptoms begin to be of concern from a diagnostic standpoint. In these instances, emotions become dysregulated as feelings of shock, disbelief, and anger persist and the griever may have repeated and inescapable images about the circumstances or consequences of the death (Shear et al., 2011). Such a reaction can result in sleep disruption, impairments in daily tasks and relational functioning, is associated with increased use of drugs and alcohol, and may manifest as suicidal thoughts and gestures (Shear et al., 2011).

There are few models, theories, and interventions specific to grief within Indigenous contexts. This lacuna has persisted despite significant experiences of grief due to loss of Indigenous territory, cultural practices, language, and elevated mortality rates. Notably, Wesley-Esquimaux (2007), writing of the impacts of colonialism on Canadian Indigenous populations, identified that colonialism rendered Indigenous people traumatized on a spiritual, physical, emotional, and psychic level, leaving a “profoundly deep and unresolved grief” (p. 8). In her analysis, she argues that “the debilitating effect of grief was the result of unremitting losses that just kept happening” (Wesley-Esquimaux, 2007, p. 8). Others have noted that within Indigenous communities, differences in religious, cultural, and social beliefs and practices may determine the degree to which grief is acknowledged, addressed, and supported by others (Kaufert et al., 1999; Preston & Preston, 1991).

## Methodology

This report draws on data from a larger study called *End of life care for Inuit living in Nunavik, Quebec* (Hordyk et al., 2016, 2017a). An ethical commitment to community-based participatory research (Hockley et al., 2013; Tuhawai Smith, 2012) in identifying EOL care concerns with an Inuit population motivated our work with local and regional partners. These included the Nunavik Regional Board of Health and Social Services, the two regional hospital centers, municipal community leaders, and school administrators.

We used a focused ethnographic methodology (Higginbottom et al., 2013; Knoblauch, 2005) combining semi-structured interviews with field notes taken during the 14 weeks of field visits between 2014 and 2015 ranging from one to six weeks duration with four communities (populations: 750 to 2,400 persons). Two of these communities were located on the Hudson coast and two were located on the Ungava coast. We also met with Montreal health care professionals engaged in care for Nunavik patients with complex palliative needs and the mortician receiving families after death. Field notes were taken by the first author to document informal discussions, the interview contexts, as well as the social and community experiences (i.e., community celebrations, festivals such as women's day at the local store, church services and funerals, visits to elder's homes, land-based excursions with Inuit, visits to homes of community members, hockey games, conversations with non-Inuit hosts, and during other local daily excursions such as grocery shopping). The primary researcher actively participated in the events when possible. These observations were not coded but rather used to contextualize findings. In total, we spoke with 103 participants in the context of health centers, schools, municipal offices, family homes, churches, and community organizations; participants included persons in health, community, education, and religious sectors. A total of 83 semi-structured in-person interviews and three focus groups, each with between five and 10 Inuit interpreters, were conducted. The majority of the interviews and all focus groups took place in employment settings and few interviews were in participants’ homes. Five Inuit participants agreed to share their views in the context of non-recorded, informal conversations. In terms of demographics, 46 participants were located on the Ungava coast, of whom 17 were of Inuit origin; 40 participants came from the Hudson coast and 28 were of Inuit origin; 18 participants were interviewed in Montreal, of whom 14 were of southern origin and four were Inuit who had relocated to Montreal. In addition to these formal discussions, we engaged in impromptu and informal dialogues with Nunavik community members during onsite visits in four communities in Nunavik. Most interviews were conducted in English or French, a second language for our Inuit participants. One interview was conducted in Inuktitut with an Inuk translator.

Transcripts of interviews and field notes written by the primary author that summarized interviews not recorded were thematically coded and an inductive analysis was performed using NVivo software (c.f. Kiger & Varpio, 2020, Nowell et al., 2017). Initial codes were generated by the first author. We then invited two Inuk reviewers to provide comments on the article and certain concepts were then clarified. Much of the data reported in this article were also included in a 2016 research report that was written for the Nunavik Regional Board of Health and Social Services. The results identified in this report were confirmed by five Inuit and non-Inuit research participants and knowledge holders. The project was approved by the Institutional Review Board at McGill University.

## Results

In this article, we focus especially on the data addressing death practices and family caregiver experiences of grief and loss. We begin with a description of the collective grief experience in the aftermath of a death. Following, we focus on beliefs related to grief expressions and the support provided to individuals and families. Finally, we conclude with reflections concerning how a community capacity approach to bereavement care may be integrated into EOL care services. Many non-Inuit health service providers with whom we spoke had never spoken with Inuit about beliefs regarding grief, attended a local funeral service, or attended to dying patients and bereaved families in their home. As mentioned, this was often due to the need to attend to other crises in the community and the fact that the training that they had access to was offered by non-Inuit and based on models in which the Nunavik realities were not factored in. In its place, some recounted myths that historically, Inuit nomads systematically left the sick, aged, and dying alone to die on the land, or that Inuit now had designated “grievers” who could do the family's grieving for them. Myths such as these minimized the profound grief-related distress common to Inuit.

### Contemporary death and burial traditions in Nunavik

Death- and grief-related beliefs and practices pre-dating the influences of colonialism and the accompanying Christian influence have been largely eradicated from Inuit communities. In Nunavik, 94% of the Inuit community identify an association to Christianity, with the large majority indicating ties to the Anglican Church and the rest affiliated to Full Gospel/Pentecostal churches (Statistics Canada, 2011). Many communities have respected community leaders or elders designated to bring news of local and more distant death to family members; these are known as *Tutsalukkaijiit* or messengers, in some dialects. These persons volunteer their time, often providing emotional and spiritual support to the family in the immediate aftermath of death. With the arrival of the internet (in 2004) and consequent access to social media such as Facebook, the role of these messengers was at times being usurped and Facebook posts regarding a death could augment the shock and suffering of the receiver.

Funeral and burial practices in Nunavik vary between communities. In some communities, nurses prepare bodies for burial. In many communities, two or three members of the church-based women's auxiliary assist a family by washing and preparing the deceased for burial. As one interpreter described, this can be a difficult job, especially in the cases of sudden and violent deaths: “it is not a fun job. It can be depressing.” She went on to explain that their local auxiliary was considering asking the health center to take responsibility for preparing the bodies of the deceased in their stead. Between the death and the burial, the body is usually kept in an indoor or outdoor morgue. Only occasionally are bodies kept in the home or church. Depending on the community or circumstances of the death, the coffin may be open or closed for visitation and the funeral service.

When Nunavimmiut die in Montreal or when the deceased is transported south for an autopsy, a Montreal funeral home prepares the body for burial after which the body is flown to the home community, usually in a simple pressed wood coffin. Family members are not permitted to touch the body before it is sent to Montreal for an autopsy. A nurse described such an instance in which a mother's suffering was compounded as she wanted to embrace her son who died suddenly in an accident but was not allowed to do so. In the case of autopsies, the funeral and burial can be delayed for days or weeks, leaving the family members in limbo not knowing when the funeral will be held, thus adding to their suffering. In the case of disappearances on land or sea, families are also kept in limbo, waiting to see if the person(s) will be found.

Men have an important role in the care of the deceased. In cases of accidental deaths and suicide, it is often men who are employed locally as first responders and firefighters who transport the bodies to the health station. Additionally, men are responsible for making coffins (usually a simple plywood design with a small cross) and operate the heavy equipment to prepare the grave for burial. Notably, there are no crematoria in Nunavik. Men also have the role of pall bearers during the funeral, a role that includes placing the coffin in the bed of a pickup to be transported to the cemetery. To our knowledge, there were no grief support services offered to these men as related to their roles. An Inuk expressed concern over this, saying, “we have to consider their mental health … it takes up a lot. It's heavy.”

The date and time of the funeral is announced on local radio. In some cases, a small pamphlet with a photo of the deceased and a written text may be given to attendees. In many communities, schools, municipal offices, and businesses, including grocery stores, are closed during a funeral. Funeral services are open to anyone who wants to attend. There are no child care options during funerals, and so babies and toddlers are also commonly seen at funerals. Young children and youth may arrive with or without family or will attend the services unaccompanied and with their peers as they would any other community event.

**Figure 1. fig1-13634615221135423:**
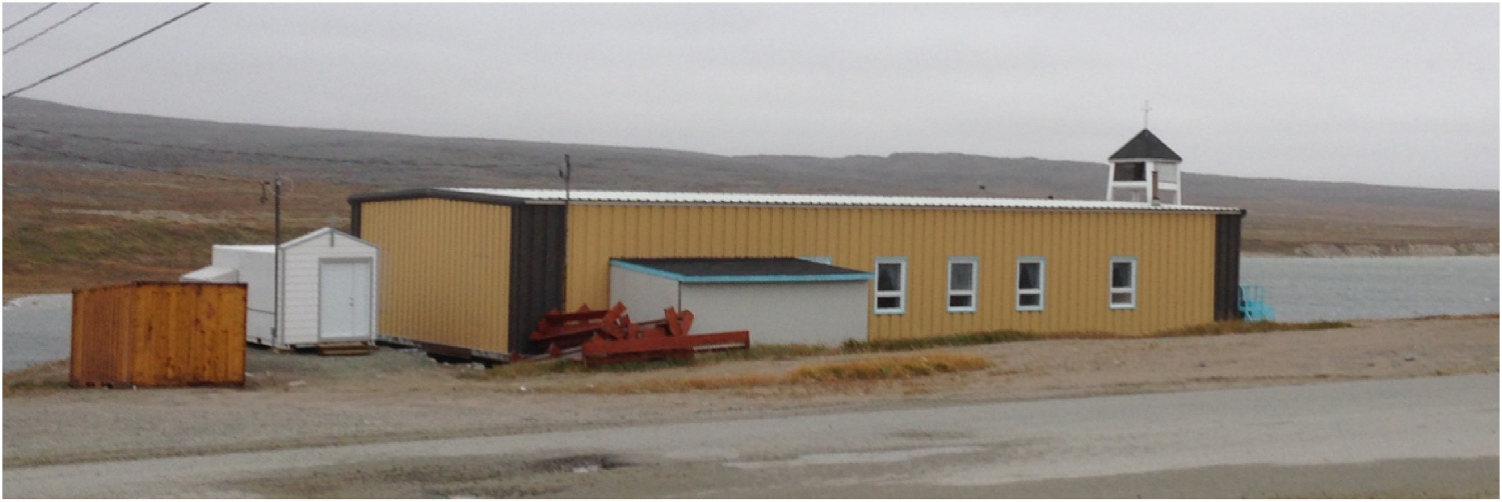
Inukjuak church with morgue (white shed on left). Source: Hordyk, permission to use.

**Figure 2. fig2-13634615221135423:**
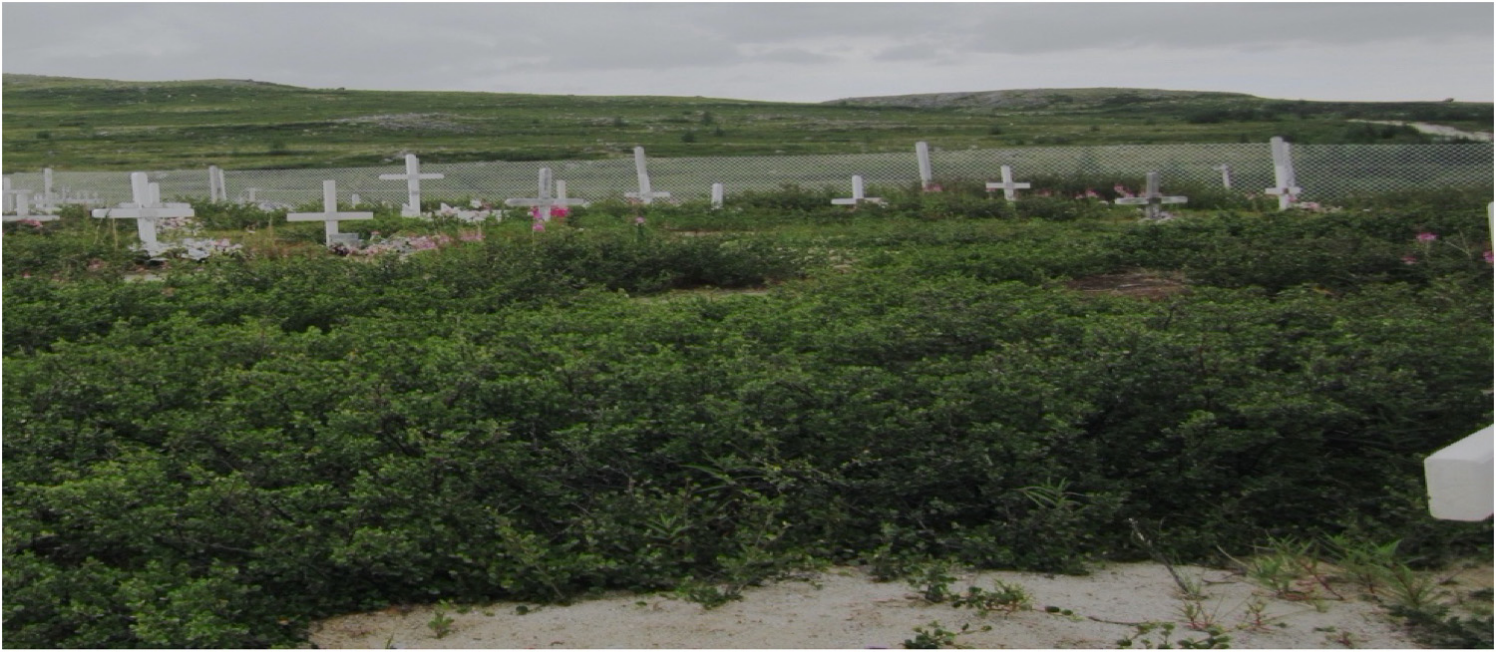
Cemetery in Nunavik 2015. Source: Hordyk.

Funeral services across Nunavik are directed by a priest or lay reader and held in the local Anglican church (see Figure 1). They follow a prescribed liturgy including scripture reading, hymns, prayers, and a religious message. Attendees are invited to share eulogies. Adults, youth, and children are invited to place flowers (usually artificial, given the climate) on the coffin at the front of the church. Attendees then line up to offer condolences to the family and proceed to the cemetery (see Figure 2) for a short song and scripture reading led by the same priest or lay reader.

#### Specific practices for death by suicide

Several Inuit participants spoke of the influence of Christian teachings on the grief and burial rituals concerning those who died by suicide. They had been taught that these deaths were unforgivable and many maintained this belief. An Inuk woman described how previously, those who died by suicide were placed vertically, rather than horizontally, in the ground, a metaphor for the belief that this person would go to hell. Others described how persons who died by suicide were buried immediately and without a time for visitation, a funeral service, or a period of public mourning. An Inuk woman recounted, “They went straight to the grave, put the land [dirt on the coffin], a little bit pray and then funeral finished.” She then reflected, “Maybe they were too much respecting their religion? ‘Cause they are just cutting their life, ‘cause we are not this way,” but she added that in her community, “This is not the case anymore. If it's a nice family, the house is full of people if someone commits suicide.” This was not true in all situations; some still opted for a quick burial.

### Grief and bereavement in Nunavik

In response to questions about the residual and cumulative impacts of grief for those providing EOL care, Inuit participants shared that historical grief-related beliefs cast a shadow over present-day practices. They recounted that, as in the past, grieving publicly after the burial was not common as many believed that this could have a negative impact on the deceased going to heaven. Though little was known about traditional grieving practices in Inuit communities, an Inuk woman described how she was told by her elders, “Don’t grieve too long. Don’t grieve for a long period of time or you stop that fire of living … that energy that you have.” Her understanding of this advice was that in small nomadic groups, family members who lost a loved one had to return to the tasks of the day, whether hunting, cooking, sewing, fishing, or establishing shelter, in order that the family could survive. There was no time to grieve. Others described historic teachings that the soul of the deceased could not proceed to the next life if the griever did not let go. Though these teachings were increasingly challenged, both Inuit and non-Inuit participants stated these teachings shape people's grief experience to this day.

#### Predeath grief practices

Grief support prior to death is manifested through concrete gestures by members of the community. These may include hunters bringing food to the home, women offering support for cooking and cleaning, and people accompanying the family by simply being present. As one person described:

… sometimes the auxiliary woman's group, they help out, and if they have to stay overnight, they select whoever wanted to do some help with the family … bring some food, bannock or bread whatever, so the patient don’t have to smell cooking in the home. Or give them support … and stay with the patient at night time … that's how Inuits are, they give a lot of support.

In addition to helping with household tasks, church auxiliary members offer spiritual and emotional support. One member described her interventions in an inpatient local health center, stating that she often accompanied the dying by singing hymns, either at their bedside or on the community radio. She translated some of the lyrics: “We have a good life in heaven, Jesus and God help us in the land. There is only one life in the world. There are two lights, going up.” She described that through her interventions, she felt the dying and their families became calmer and less afraid of death because they felt they were being helped by God.

#### Grief practices between time of death and burial

A community member described how in certain instances, elders asked to be buried immediately following their death. She explained that they want to be at peace when they go, that they are done with the physical world and want to go to the spirit world and that an immediate burial would hasten this process. Most described, however, that during the time between the death and burial, family and community members may visit the family at their home, sharing food, stories, silence, tears, and laughter. Some communities hold formal visitation for families and community members in the church before the funeral. Others hold visitations in the nursing station or the morgue (in most communities, this is a small walk-in refrigerated cabin located near the church or health station). In some cases, there are no opportunities for visitations that include the viewing of the body. A participant described her concern about this shift, stating that her grandmother would say, “you have to see the body dead to start the grief process.” Now she felt that people deny the reality of death more than in the past.

The tradition of keeping vigil with the family until the funeral is changing. One Inuk participant stated, “the lifestyle of work and family does not give room to visit like before. It’s late at night before people have free time.” Another Inuk man attributed this to a transition in communities from collective practices where members looked out for each other and spent leisure time together, toward individualism in which people are more pre-occupied with their own needs and spend more time with television and Facebook.

### Post-burial grief practices

The belief that grieving must end with the burial service was prevalent among members of the four communities that we visited. One Inuk woman stated that people are usually very helpful prior to and during the funeral, bringing food and making sure the funeral service and burial run smoothly, but are less present afterward to accompany the grieving; “People are very good about that up north I find … but once the body's buried, it's like they’ve done their part; that's it.” Another explained that this reticence may be rooted in traditional teachings: “There is a myth about people who grieve too long … that ‘I’m trying to get to heaven and will not make it to heaven because you’re crying too much’; the person's tears will make the stairs slippery.”

One Inuk participant summarized how expressions of grief are shifting for many Nunavimmiut today. She used the term “in-between generation” to describe how people were transitioning from old to new ways of coping with loss. The new ways allow for more expression of emotion and conversation concerning the deceased. A woman speaking of the death of a child noted that after the funeral, “that's the time when you are starting to feel that you don’t have the person anymore. That's when you needed to be heard I guess, ‘cause when you talk about it, it becomes easier.” An Inuk community worker discussed the loss of her grandfather and challenged what she had been taught:

Here in the community, they say when somebody passes away, for them to rest in peace, you have to just let them go. But for some, it's very hard to just let someone you love go … you don’t need to let go of someone who passed away.

Some families now engage in collective practices to commemorate a death after the funeral. As one Inuk participant described:

After something tragic happens in their family or they’ve lost a family member … a lot of them go to the land. When they are on the land, they do a lot of healing … just being in a tent, remembering their memories of their loved one.

She also stated that some may increase church attendance after a death as singing and praying in a collectivity may provide an emotional release. The option to go out on the land is not possible for all families, however, because of increasing costs associated with the supplies and transportation needed for such excursions, such as the price of all-terrain vehicles, snowmobiles, and boats, as well as cost of fuel and maintenance.

One reason cited for these changing grief practices was Canada's Truth and Reconciliation Commission (TRC) held between 2008 and 2015. Inuit had been forcibly separated from their families and placed in residential school facilities often far from home for a part or the duration of their childhood (TRC, 2015). Many school attendees had been separated from their families, had been forced to renounce their culture and language, and suffered lifelong consequences. During the TRC hearings, a team of four Inuit women, known as the Inuit Values and Practices Team, was convened to travel to Nunavik communities to facilitate group healing opportunities for survivors. According to members of this team, these group sessions and hearings opened the door to publicly acknowledge grief as an ongoing process and to recognize that grief can be rooted in the long-term effects of the residential schools and other harmful colonial practices. In addition, several Nunavimmiut were invited to provide or witness testimonies alongside of members of other Indigenous communities in the context of public hearings held in April 2013 in Montreal, Quebec. This collective sharing of grief, accompanied by emotional support services rooted in Indigenous values and traditions, contributed toward allowing persons to acknowledge long-term grief.

In 2019, the Canadian government alongside several Inuit-serving organizations, including Inuit Tapiriit Kanatami, launched the *Nanilavut Initiative* (*Nanilavut* translated as “let's find them”). Their database includes information concerning 4,500 Inuit who were displaced for medical treatment during the height of the tuberculosis epidemic between the 1940s and 1960s. They strive to offer information concerning where individuals were treated, died, and were buried, though this information is not always available. They also provide funds for travel for Inuit family members to visit the burial sites of relatives, commemorative activities, gravesite enhancement funds, and health support if desired.

#### Formal grief support

Professional trainings, conferences, and social media now accessible to Nunavimmiut are also currently conveying the idea that grief is an ongoing process. One Inuk counselor described this evolution in her understandings of grief: “Today, we say when you are grieving someone, feel the pain, acknowledge it; let somebody know that you are hurting.” Another community worker related how during a professional conference, she had responded to an invitation to grieve the loss of a loved one who had died several years prior. Upon returning to her community, she shared her new awareness of extended grief processes on the local radio.

Both the *Nunavik Regional Board of Health and Social Services* and *Kativik Ilisarniliriniq*, the Nunavik school board, have begun to offer occasional grief trainings to direct services providers such as school counselors, wellness workers, social workers, and suicide prevention workers. The Inuit Values and Practices team, who received training and support largely in exchanges with Indigenous and non-Indigenous persons at Health Canada, are now available to visit families who are grieving a death due to accident or suicide. However, they have stated that the need far exceeds their capacity. In addition, the Nunavik Regional Board of Health and Social Services coordinates a region-wide suicide prevention conference, *Puttautiit*, with presentations and healing sessions and workshops offered by Indigenous persons from within and outside of Nunavik. Inspired by the *Dialogue-for-Life* conference offered annually by the First Nations and Inuit Suicide Prevention Association of Quebec, the conference objectives are public awareness, healing opportunities for communities, and support and training for Inuit engaged in social services.

*Kativik Ilisarniliriniq* offers occasional grief education and awareness sessions to local school counselors and has published a grief support guide and children's book on grief in Inuktitut and English. In addition, across Nunavik, people with experience in education, social services, spiritual care, and leadership are being contracted to offer support and training in their communities concerning topics such as decolonization, grief support, and counseling skills. Non-Inuit social workers, psychologists, and psychiatrists are also providing grief support. However, time constraints, language barriers, cultural differences, and geographic distance are significant limiting factors in their abilities to offer care.

#### Informal grief support

Informal networks of family and friends in communities also provide support to the bereaved after the funeral has ended. Nunavimmiut concerned about grief and the healing process can be heard on the radio, seen on Facebook, and in community and church gatherings, spontaneously sharing their stories of loss and passing along messages and resources. Inuit support counselors travel between communities to offer what are termed “healings,” which include Bible reading and prayers for the specific purpose of alleviating suffering, including grief.

In spite of these efforts, participants were clear that existing formal and informal support structures were not adequate to address the bereavement needs of Inuit in Nunavik. Local and regional grief support persons are often overburdened by demands. Lack of personnel and resources meant that often grieving individuals and families have no one with whom to speak. Confidentiality was an ongoing issue, as it can be difficult to maintain in small communities where family and friendship networks are intertwined. Inuit school counselors, wellness workers, and social support workers noted that they had limited training in grief counseling and were often too busy dealing with immediate crises in their work to have time for grief support interventions. This was also true for non-Inuit social workers in communities; in addition to not speaking Inuktitut, they were uncertain about cultural grief practices and their role in supporting families. There are also high turnover rates in these positions.

### The burdens of grief

Though there are no official statistics available to identify the numbers of unexpected deaths in each community, the sheer amount of loss described by Inuit participants was described as a barrier to adequately grieving. Families and community members are intricately interwoven and, as such, everyone is personally affected by all deaths in their extended community. As one Inuk put it, “People do not have time to grieve. There are too many deaths.” These multiple grief experiences were described as “a never-ending experience of grief that was like forever.” The consequences are important, leading a school administrator to state that this accumulation of grief and lack of resources to address it meant that “people go a little crazy after a while,” crazy being a term that she used to indicate a high level of distress. We spoke with an Inuk counselor who herself felt inadequate to support students and related this to her own struggles as a mother to respond to the needs of her teenage son who had lost four classmates to suicide.

Those who had lost family members or friends to suicide could be socially alienated by the traumatic circumstances of the death, the ambivalent support responses of community members, and by their own feelings of shock, anger, and guilt. An Inuk counselor described that when people experienced loss due to suicide or another kind of violent death, “they have more bitterness and uneasiness with themselves … this can be toxic.” A father who had lost his son to suicide described how in the aftermath, each family member isolated themselves and the loss touched each of them differently: from the siblings who were the first to find their brother, to the mother and father, each with their unique relationship and, consequently, their own interpretation of his actions.

Importantly, many Inuit participants spontaneously contextualized their grief experience within historical accounts of family and community losses due to colonial acts of violence. The grief that they felt from the terminal illness and death of a loved one was inseparable from these histories and the family trauma that had resulted. Several participants recounted being forcibly separated from their parents for months or years when placed in residential schools or in hospitals with a diagnosis of tuberculosis. Some described forced relocations to communities where they were placed in villages with clans with whom they were either unfamiliar or adversarial and who had different dialects and customs. Livelihoods were threatened when they were placed far from their familiar hunting and fishing grounds. Some described the mass slaughter of their sled dogs, sources of transportation between summer and winter camps and of access to food sources, many having been bred by families for generations.^1^ Reference was made to the loss of personal and cultural identities following these traumas and the intergenerational impact perpetuating the suffering. An Inuk educator observed that because this suffering was not explicitly the result of a death, per se, the accompanying feelings of grief and loss were often not recognized or openly acknowledged in communities as legitimate reasons for grieving.

## Discussion

While seeking to understand the strengths and resources within Nunavik's Inuit communities related to EOL care, we realized how palpable grief experiences are for family caregivers in Nunavik. Their grief related to the ongoing dying of family members with cancer, respiratory illness, and other terminal illnesses was compounded by multiple prior and current loss experiences, including those related to colonial policies and the alarming number of deaths due to suicide and accidents in local communities. These accumulated loss experiences have had lasting and intergenerational impacts. While support is evolving to keep up with the needs of the bereaved, our data suggest that experiences of loss continue to be overwhelming for many in Nunavik.

Our findings suggest that the lack of grief support is due in part to the sheer numbers of persons working in health, social, and education settings that are themselves grieving multiple deaths, as well as the lack of regular and culturally appropriate grief support training and financial resources that could equip more persons to be offering bereavement care in their own communities and language. To further the development of these services, we have documented conversations with research participants regarding the grief experience and associated beliefs, and have contextualized this information with our own observations of grief practices. As Canada has several Indigenous groups, each with their own traditional belief practices surrounding the grief experience and within which there are significant differences between how families and communities grieve, we suggest that a deeper understanding of the grief context in Nunavik in particular is needed.

Current theoretical and practice frameworks concerning the prolonged and complex grief experience do not adequately address the long-term grief-related suffering experienced by many of the Inuit providing EOL care. Though not exhaustive, our study suggests that these models do not take into account key elements, such as language, culture, Indigenous expertise, and current community practices. Nor do they consider the collective nature of traumatic and prolonged manifestations of grief in which significant numbers of premature deaths impact large networks of families and communities. We propose that further steps need to be taken to identify both the common and the unique aspects of the Nunavik grief experience and of how to increase support services.

Our findings corroborate a wider tendency for language and culture to be omitted in bereavement care (Breen et al., 2017). A community capacity approach is needed, which identifies, confirms, and supports the assets that people have for caring for themselves and others in bereavement (Rumbold & Aoun, 2014), and that recognizes Inuit as the true experts in their care. Health institutions may be associated with colonial abuses of power (Maddocks & Rayner, 2003), and communication challenges may arise due to linguistic and cultural differences (Kelly et al., 2009). Inuit living in Nunavik are caught in an intersection of multiple forms of oppression (Crenshaw, 1991; Hill Collins, 1998) in which hierarchies based on ethnicity, skin color, Western institutionalized forms of education, social class, sex, income, linguistic capacities, and physical capacity determine whose knowledge is recognized and who has decision-making power in health and social care. These power differentials and their impact on care in Nunavik and elsewhere were recently highlighted in a public inquiry examining the relationships between Indigenous communities and public services in Quebec (Viens Commission, 2019).

A community capacity-building approach specific to bereavement care necessitates remaining attentive to individual and organizational prejudices and biases. This entails engaging with Inuit service providers and the bereaved through research based on relationships, trust, humility, and accountability (Kilian et al., 2019). These exchanges include dialogue (Breen et al., 2017) with the recognition that direct translation is impossible and that meanings may be lost in the process. Within communities themselves, differences in religious, cultural, and social beliefs and practices may determine the degree to which the grief of caregivers is acknowledged, addressed, and supported by others (Kaufert et al., 1999; Preston & Preston, 1991).

This study suggests that grief support services and training for Inuit already working in social, health, education, and civic institutions in Nunavik are needed so they may be better positioned to address grief in the context of their workplace and community. A triage model that identifies those with risk factors predisposing them to prolonged grief and related suffering may be useful (Aoun et al., 2012). As the Inuit Values and Practices team recounted, effective training can take place in the context of knowledge exchanges between Inuit and non-Inuit. Here, persons from diverse Inuit communities share of their experiences, knowledge, and educational models in order to co-develop grief support strategies congruent to Inuit. In doing so, more people will be equipped to offer training and support in their own language. As the following discussion suggests, these knowledge exchanges between Inuit and non-Inuit must be based on relationship building and cultural decentering.

Nunavimmiut engaged in community mobilization efforts are hindered by the sequelae of historic and contemporary manifestations of colonialism (Fraser et al., 2019). Quijano (2007) describes how colonialism as a systematic repression robbed persons outside of the Western system of “modes of knowing, of producing knowledge, of producing perspectives, images and systems of images, symbols, modes of signification” rooted in their own cultures and tradition, and replaced these with beliefs and images of the dominators. Wesley-Esquimaux (2007) proposes that Indigenous healing entails “a recovery of awareness, a reawakening to the senses, a re-owning of one's life experience, and a recovery of people's enhanced abilities to trust this experience” (p. 10). This “reawakening” requires certain conditions to be in place.

For research and practice to be pertinent and for the development of grief interventions to be effective, a perspective that welcomes Inuit language, symbols, images, and practices, as well as local forms of knowledge transmission, is essential to recognize and build on protective factors already in place. In addition, knowledge can be gleaned about formal and informal grief healing practices. This process requires relationship building as a part of learning in the context of educational, health, and social service provision (Gabriel et al., 2019) and associated research (Bessarab & Ng'Andu, 2010). On the other hand, community members are invited to critically examine existing non-Inuit models, transforming what is pertinent into their own language, social, and cultural contexts. Such a commitment entails allocating financial resources for these relational and theoretical exchanges to take place.

## Limitations

An important limitation in this study was language: for all but one elder, Inuit were interviewed in their second language of French or English. Mastery of this language varied widely, thus rendering it difficult for some to express themselves with the nuance that the topics of death and grief warrant. In addition, several English or French terms could not be directly translated into Inuktitut. We do not know to what degree Inuit participants adapted their intended meanings to translate Inuktitut terms and concepts into English or French. The interpreter also faced this challenge explaining for example that the words “grief” and “forgiveness” were not the exact words of the elder in the interview but concepts that were described in the context of his specific story that she translated using these terms. That said, due to the sensitive nature of the conversations concerning grief and death, we chose not to pursue the more explicit discussions of terminology with the interviewees. In future studies, it would be pertinent to explore this question of terminology and translation with Inuit who are not simultaneously speaking of their personal grief and loss experiences. Second, we conducted this study in four of Nunavik's larger communities and thus could not determine how EOL care or the associated grief experience may differ in smaller communities of only a few hundred persons.

## Conclusion

This ethnographic study of grief and mourning practices in Nunavik aimed to highlight the bereavement experience of Inuit providing EOL care and the strengths and capacities already present in communities to address this grief, as well as to identify future areas of research that can lead to better support of families and communities. To accomplish this, conceptual models concerning prolonged and traumatic forms of grief and their associated intervention must be reexamined in the light of the bereavement experience of Inuit. Models need to recognize compound grief experiences (due to many tragedies experienced by tight networks of family and community members), the collective nature of the bereavement experience, and grief emerging from historic and contemporary manifestations of colonialism. Exchanges of knowledge between Inuit and non-Inuit, cognizant of the hierarchies of oppression inherent in health, social, educational, and political institutions, and which recognize Inuit as the ultimate experts in their care, are pertinent to furthering this work.[Fig fig1-13634615221135423][Fig fig2-13634615221135423]

## Notes

1. See video-recorded testimonies from the Qikiqtani truth commission (2007 to 2010) for more information about the impact of these historical events on Inuit families: https://www.qtcommission.ca/index.php/en
